# Effects of Cellulase and *Lactiplantibacillus plantarum* on the Fermentation Parameters, Nutrients, and Bacterial Community in *Cassia alata* Silage

**DOI:** 10.3389/fmicb.2022.926065

**Published:** 2022-07-07

**Authors:** Zhenyu Xian, Jiaqi Wu, Ming Deng, Meng Wang, Hanchen Tian, Dewu Liu, Yaokun Li, Guangbin Liu, Baoli Sun, Yongqing Guo

**Affiliations:** College of Animal Science, South China Agricultural University, Guangzhou, China

**Keywords:** *Cassia alata*, cellulase, *Lactiplantibacillus plantarum*, silage quality, microbial community

## Abstract

Silage *Cassia alata* (CA) can alleviate feed shortage in some areas to a certain extent and reduce feed costs. The present research evaluated the effect of cellulase (CE) and *Lactiplantibacillus plantarum* (LP) on the fermentation parameters, nutrients, and bacterial community of CA silage. Chopped CA was ensiled with three different treatments, namely, no inoculant (CK), CE, and LP, and the indexes were determined on the 2nd, 6th, 14th, and 30th days of silage fermentation. The fermentation parameters indicate that the pH value of the three groups decreased and then increased with the ensilage process, and the lowest value was observed on the 14th day. The CK and LP groups attained the highest value on the 30th day, while the CE group attained the highest value on the 2nd day. Additionally, the pH value and NH_3_-N content were significantly lower (*P* < 0.05) in the CE and LP groups than in the CK group. In terms of nutrients, crude protein (CP) contents significantly increased (*P* < 0.05) in the CE and LP groups on the 30th day. The neutral detergent fiber (NDF) and acid detergent fiber (ADF) contents of the CE group were significantly and negatively associated with fermentation time, and the water-soluble carbohydrate (WSC) contents of the three groups were significantly lower during ensiling. In comparison with the CK group, the NDF and ADF contents were significantly reduced (*P* < 0.05), and the WSC content increased (*P* < 0.05) in the CE group on day 30. Sequencing analysis of bacterial communities showed that *Lactobacillus* became the most dominant genus in the ensilage process. Moreover, both CE and LP groups increased the abundance of *Lactobacillus* and decreased that of *Klebsiella, Weissella*, and *Acetobacter* in comparison to the CK group, in which LP had a better effect. CE and LP could further improve the silage quality of CA, and LP had a more significant effect in reconstructing the bacterial community in the silage environment.

## Introduction

With the rapid development of animal husbandry, the shortage of high-quality silage is becoming increasingly serious. Thus, the development of new high-quality silage is essential. *Cassia alata* (CA), a major species of the *Leguminosae* family that originated from tropical South America, is cultivated in Yunnan and Guangdong provinces in China with a large plantation area and high yield. The fast growth, tolerance to barrenness, strong adaptability, and high protein content (Lim, 2014) of CA indicate its value in the development of feed. Although CA is mainly used for ornamental and medicinal purposes, it has also been used in feed. CA might have difficulty achieving satisfactory silage quality as a *Leguminosae* plant mainly because of the low content of water-soluble carbohydrate (WSC) and insufficient counts of epiphytic lactic acid bacteria (LAB). Therefore, the adverse effects should be addressed to improve the nutritional value and to obtain high-quality silage.

Considering the shortage of the low content of WSC in fresh CA, cellulase (CE) can be added. CE is widely recognized as the most commonly used enzyme for its applications in forage preservation (Hu et al., 2021). CE is a cell-wall degrading enzyme that can increase the rate of cell-wall degradation in plants and decrease the neutral detergent fiber (NDF) and acid detergent fiber (ADF) contents of silage by cleaving β-(1,4)-linkages in cellulose to release polysaccharides and then release glucose (Nadeau et al., 2000). Considering that glucose is the substrate for LAB fermentation, CE can indirectly promote LAB fermentation (Mu et al., 2020). Besides, the application of CE is beneficial for ensiling low-WSC forages. CE promotes fiber hydrolysis and releases glucose, which enables the multiplication of LAB and the production of lactic acid (LA), remarkably improving fermentation parameters and increasing the abundance of LAB in mixed silages of soybean residue and corn stover (Zhao et al., 2021). In addition, CE addition increases the content of true protein and decreases the fiber and NH_3_-N contents of mulberry leaf silage (He et al., 2019).

In addition to improving the content of WSC in fresh CA to obtain high-quality silage, the counts of epiphytic LAB can also be increased. The homofermentative LAB, such as *Lactiplantibacillus plantarum* (LP), is the most used fermentation prompter for silage production. LP can rapidly and effectively metabolize the WSC into LA, thus reducing the pH to 4 (Yitbarek and Tamir, 2014; Mu et al., 2020) and promoting silage fermentation. Low pH value and anaerobic conditions can inhibit the activity of undesirable microbiota and the inherent hydrolysis of plant protein, thereby reducing the production of NH_3_-N (Chen et al., 2020a). In the silage production, some spoilage bacteria reduce the quality of alfalfa silage, making the effect of silage less than expected. LP can rapidly eliminate pathogens under adverse conditions and improve the quality of silage (Ogunade et al., 2016). Adding LP reduces the pH value and the contents of acetic acid (AA), propionic acid (PA), and NH_3_-N in king grass silage. LP changes the bacterial community and increases the abundance of the desirable members of the *Lactobacillus* genus (Zi et al., 2021). In addition, the bacterial species of LP are considered by European Food Safety Authority to be suitable for the qualified presumption of safety approach (Additives EPo, 2021).

Harvesting CA for silage can alleviate feed shortage in some areas to a certain extent and reduce feed costs. Therefore, extending the use of CA in feed is essential for the diversification of the CA industry and meeting the rapid development of high-quality silage for ruminants. However, the microbial composition of CA silage and the reaction mechanism of additives remain unclear. The effects of CE and LP on the diversity of CA silage quality and microbiota need to be studied. However, CE and LP have beneficial effects on the fermentation and microbes of CA silage. This hypothesis was tested by assessing the effects of CE and LP on the fermentation parameters and microbial diversity of CA silage in the current research.

## Materials and Methods

### Raw Materials and Silage Preparation

The whole plant CA in the blooming stage with a height of 1.2 m (with a stubble height of 30 cm) was harvested and chopped into 10–20 mm lengths by using a grass shredding machine on September 27, 2020 from the ecological breeding farm of the Guangshunhai Food Limited Company (116°133′N; 24°124′E, Meinan Town, Meixian District, Meizhou City, Guangdong Province, China). The raw material was used to determine the chemical composition. The chopped CA was divided into three groups and ensiled as follows: (1) CK group (control group without additives; sprayed with 1 g of distilled water), (2) CE group (Guangdong VTR Bio-Tech Co., Ltd., China mainland; mainly including C_1_-CE, C_X_-CE, and β-(1,4)-glucosidase; CE activity as 5 × 10^5^ U/g; addition amount: 0.1 g/kg of fresh material (FM) dissolved in 1 g of distilled water), (3) LP group (LP application rate: 1 × 10^6^ cfu/g FM suspended in 1 g of distilled water; No. 23490, a native strain isolated and selected from stylo silage based on its acid productivity as described by Wang et al., 2019a). After mixing CA with CE and LP thoroughly, approximately 200 g of silage material was placed into fermentation bags (20 × 30 cm) with a one-way breather valve, vacuum-sealed immediately, and then stored at room temperature (25 ± 3°C). Three replicate bags were prepared for each treatment, and four samples were uniformly obtained from each bag on days 2, 6, 14, and 30 of ensiling. The first sample was used to test the parameters of silage fermentation, the second one was dried (65°C for 48 h) and passed through a 1-mm sieve in a grinder for chemical composition analysis, the third one was used for microbial counts, and the fourth one was used for the microbial diversity analysis.

### Fermentation Parameters Analysis

As described by Wang et al. (2019a), 5 g of sample of the silage was mixed with 45 mL of distilled water, stored at −4°C for 24 h, and filtered to obtain the extract to determine their fermentation characteristics. The pH was directly measured using a pH meter (Sartorius PB-10). Ammonia-N (NH_3_-N) content was determined using phenol-sodium hypochlorite colorimetry (Ke et al., 2017). LA was detected using the p-hydroxybiphenyl colorimetric method (Barker and Summerson, 1941). Volatile fatty acids, such as acetic acid (AA), propionic acid (PA), and butyric acid (BA), were analyzed using high-performance gas chromatography (Agilent 7890B). The vaporization chamber parameters are as follows: carrier gas, N_2_; split ratio, 40:1; injection volume, 0.4 μL; and temperature, 220°C. The column parameters are as follows: HP-INNOWax capillary column constant flow mode; flow, 2.0 ml/min; and average linear velocity, 38 cm/s. The column thermostat parameters are as follows: programmed heating 120 °C (3 min)-10 °C/min−180 °C (1 min). The detector parameters are as follows: H_2_ flow rate, 40 ml/min; airflow rate, 450 ml/min; column flow rate makeup air flow rate, 45 ml/min; FID temperature, 250 (Shao et al., 2005).

### Nutrient Analysis

The removed samples were oven-dried at 65°C for 48 h to calculate the dry matter (DM), then crushed and passed through a 1-mm sieve for nutrient analysis. The results are expressed as DM. According to the methods of the Association of Official Analytical Chemists (Association of Official Analytical Chemists, 1990), crude protein (CP) was determined using a Kjeldahl nitrogen analyzer (Kjeltec 2300 Auto Analyzer, FOSS Analytical AB). Van Soest et al. (1991) measured NDF and ADF using an automatic fiber analyzer (ANKOM 2000i). The content of WSC was determined using the anthrone-sulfuric acid colorimetric method (Owens et al., 1999).

### Microbiological Analysis

The trial procedures described in this section were similar to those previously reported (Wang et al., 2020a). Briefly, the silage was unpacked and sampled under aseptic conditions and subjected to microbiological analysis by plate counting. One portion of 5 g silage samples was homogenized with sterilized saline (45 mL) in an orbital shaker for 30 min, and the supernatants were serially diluted from 10^−1^ to 10^−6^. LAB and coliform bacteria (CB) were separately cultivated on Man Rogosa Sharpe agar and Violet Red Bile agar at 30°C for 48 h in an aerobic incubator. Yeasts and molds were cultured in Rose Bengal agar following incubation at 28°C for 48 h.

### Bacterial Community Sequencing Analysis

The bacterial community of silage was analyzed using high-throughput sequencing at the Guangzhou Genedenovo Biotechnology Co., Ltd. Microbial DNA was extracted from silage samples using the HiPure Soil DNA kit (Magen, Guangzhou, China) in accordance with the kit's instructions. The final DNA purification was determined using the NanoDrop 2000 microspectrophotometer (Thermo Fisher Scientific, USA), and the DNA integrity was assessed by 2% Biowest Agarose gel electrophoresis. Thereafter, the 16S rDNA V3-V4 regions of the ribosomal RNA gene were amplified using PCR using the primer pairs of 341F (5′-CCTACGGGNGGCWGCAG-3′) and 806R (5′-GGACTACHVGGGTATCTAAT-3′). After amplification, amplicons were purified using the AxyPrep DNA gel extraction kit (Axygen Biosciences, Union City, CA, USA) according to the manufacturer's instructions, and quantified using the ABI StepOnePlus real-time PCR system (Life Technologies, Foster City, USA). Then, sequencing was performed on the PE250 mode pooling of Novaseq 6000.

High-quality clean reads were obtained by filtering raw reads according to the rules using FASTP (version 0.18.0). Then, paired-end clean reads were merged as original tags using FLSAH (version 1.2.11) with a minimum overlap of 10 bp and a mismatch error rate of 2%. The noise sequences of the original tags were filtered under specific filtering conditions to obtain high-quality clean tags. The clean tags were clustered into operational taxonomic units (OTUs) of ≥97% similarity using the UPARSE (version 9.2.64) pipeline. All chimeric tags were removed using the UCHIME algorithm, and effective tags were obtained for further analysis. The tag sequence with the highest abundance was selected as the representative sequence within each cluster. The representative OTU sequences were classified into organisms by using a naive Bayesian model with the RDP classifier (version 2.2) based on the SILVA database (version 132) at a confidence threshold value of 0.8 to obtain taxonomic classification at the phylum, class, order, family, and genus levels. Alpha diversity was analyzed using QIIME (version 1.9.1). Beta diversity was analyzed in the R project Vegan package (version 2.5.3). The functions of microflora were predicted based on the Tax4Fun (version 1.0) database.

### Statistical Analysis

Fermentation parameters, chemical characteristics, microbial population, and alpha diversity index data were analyzed using a two-way analysis of variance (ANOVA) in the IBM SPSS 25.0. The model for data processing is as follows:


$$\begin{array}{l} Y_{ij}=\mu +\alpha_{i}+\beta_{j}+\alpha \beta_{ij}+\;\varepsilon_{ij}, \end{array}$$

where *Y*_*ij*_ is the variable of interest, μ is the overall mean, α_*i*_ is the fixed effect of ensiling days, β_*j*_ is the fixed effect of different additions, αβ_*ij*_ is the interaction of ensiling days and additions, and ε_*ij*_ is the residual error. Duncan's multiple comparisons were used to examine the significant differences between various treatments (*P* < 0.05).

## Results

### Characteristics of Fresh *Cassia alata* Before Ensiling

The chemical compositions of fresh CA before ensiling are listed in Table 1. The contents of DM, CP, NDF, ADF, and WSC were 22.82, 18.89, 64.37, 38.94, and 3.35%, respectively. Fresh CA has high NDF and ADF content and low WSC.

**Table 1:** Nutrients of fresh *Cassia alata* prior to ensiling (±SD, *n* = 3).

**Item**	***Cassia alata***
Dry matter (% AF)	22.82 ± 0.45
Crude protein (% DM)	18.89 ± 0.05
Neutral detergent fiber (% DM)	64.37 ± 0.30
Acid detergent fiber (% DM)	38.94 ± 0.15
Water soluble carbohydrate (% DM)	3.35 ± 0.04

*DM, dry matter; AF, as fed*.

### Dynamics of Fermentation Parameters in *Cassia alata* Silage

Table 2 shows the dynamics of fermentation parameters in CA silage. At each measurement time, the pH values in CE and LP groups were significantly lower (*P* < 0.05) than those in the CK group. With the increase of ensilage time, the pH values of each treatment group decreased and then increased, reaching the lowest on day 14. Among the samples, only the pH value in the CE group was below 4.2 on days 14 and 30. Besides, when the silage was fermented for 30 days, the pH values of the CE group were significantly lower (*P* < 0.05) than those of the CK and LP groups. The NH_3_-N content of the CK, CE, and LP groups increased significantly (*P* < 0.05) throughout the ensilage period. The NH_3_-N contents of the CE and LP groups were significantly lower (*P* < 0.05) than those of the CK group. The NH_3_-N content of the LP group was significantly lower than those of the two other groups (*P* < 0.05) after 30 days of fermentation, while the contents of NH_3_-N in the CK group were the highest. The LA content of each group increased (*P* < 0.05) during fermentation, and the LP group had the highest LA content on day 30. The AA content of each group increased significantly with the increase of silage days (*P* < 0.05) and reached the highest on day 30, where the CE group had the highest content and the LP group had the lowest content. On day 2 of ensilage, PA was not detected in the silage of each treatment group. After day 2, the contents of PA in each group increased significantly with fermentation (*P* < 0.05), but no significant differences (*P* < 0.05) were observed among the groups. The BA content of the CK group increased significantly (*P* < 0.05) with the increase of ensilage days. The BA content of the CK group was significantly (*P* < 0.05) higher than that of the CE and LP groups on day 30.

**Table 2:** Fermentation parameters of ensiled *Cassia alata*.

**Item**	**Treatment**	**Ensiling days**				**SEM**	**Significant**		
		**2**	**6**	**14**	**30**		**Days**	**Treatments**	**Days × Treatments**
pH	Control	4.61^aB^	4.59^aBC^	4.52^aC^	4.74^aA^	0.009	^**^	^**^	^**^
	Cellulase	4.49^bA^	4.30^bB^	4.16^cC^	4.18^cC^				
	*Lactiplantibacillus plantarum*	4.28^cB^	4.24^bB^	4.22^bB^	4.57^bA^				
NH_3_-N (g/kg DM)	Control	0.0098^aC^	0.025^aB^	0.028^aAB^	0.034^aA^	0.006	^**^	^**^	ns
	Cellulase	0.0086^abC^	0.018^abB^	0.023^abAB^	0.028^aA^				
	*Lactiplantibacillus plantarum*	0.0069^bC^	0.012^bB^	0.017^bA^	0.016^bA^				
Lactic acid (g/kg DM)	Control	79.13^C^	93.65^B^	98.65^AB^	104.73^aA^	0.648	^**^	ns	ns
	Cellulase	81.90^B^	97.43^A^	92.51^A^	99.62^bA^				
	*Lactiplantibacillus plantarum*	82.00^C^	94.37^B^	94.70^B^	106.10^aA^				
Acetic acid (g/kg DM)	Control	9.93^aD^	18.22^aC^	27.60^aB^	30.68^aA^	0.153	^**^	^**^	^**^
	Cellulase	8.85^aD^	14.71^bC^	22.60^bB^	32.56^aA^				
	*Lactiplantibacillus plantarum*	6.22^bD^	10.63^cC^	15.19^cB^	23.29^bA^				
Propionic acid (g/kg DM)	Control	ND	0.45^B^	1.78^A^	2.41^A^	0.073	^**^	^*^	ns
	Cellulase	ND	0.36^B^	1.18^A^	1.63^A^				
	*Lactiplantibacillus plantarum*	ND	ND	0.67^B^	2.00^A^				
Butyric acid (g/kg DM)	Control	ND	0.40^C^	1.49^B^	5.09^aA^	0.073	^**^	^**^	^**^
	Cellulase	ND	ND	ND	0.63^b^				
	*Lactiplantibacillus plantarum*	0.24	0.61	0.57	1.03^b^				

*ND, not detected; SEM, standard error of means*.

* *P < 0.05*;

** *P < 0.01; ns, no significant effect*.

*Values in the same row (^A−C^) or column (^a−c^) followed by different letters differ at P < 0.05*.

### Dynamics of Nutrients in *Cassia alata* Silage

The nutrient analysis results are displayed in Table 3. The difference in DM contents among the three groups was insignificant (*P* > 0.05). The contents of CP in the CE and LP groups were significantly higher than those of the CK group on day 30 (*P* < 0.05). The contents of NDF and ADF in the CE group decreased significantly (*P* < 0.05) with the increase in fermentation time. In addition, the contents of NDF and ADF in the CE group significantly (*P* < 0.05) decreased compared with the CK group on day 30. In comparison with fresh CA, the contents of NDF and ADF in the CE group decreased to 34.27 and 28.09%, respectively, on day 30. The contents of WSC significantly (*P* < 0.05) decreased in the three groups during ensilage and were significantly (*P* < 0.05) higher in the CE group than in the CK and LP groups on day 30.

**Table 3:** Nutrients of ensiled *Cassia alata*.

**Item**	**Treatment**	**Ensiling days**				**SEM**	**Significant**		
		**2**	**6**	**14**	**30**		**Days**	**Treatments**	**Days × Treatments**
Dry matter (% FM)	Control	22.27	19.98	21.89	21.57	0.398	ns	ns	ns
	Cellulase	20.97	20.79	21.20	21.06				
	*Lactiplantibacillus plantarum*	22.54	22.06	21.84	25.88				
Crude protein (% DM)	Control	17.33^bA^	14.82^cC^	15.60^bB^	12.22^bD^	0.051	^**^	^**^	^**^
	Cellulase	16.74^b^	16.40^b^	16.44^a^	15.83^a^				
	*Lactiplantibacillus plantarum*	18.88^aA^	17.75^aB^	16.67^aC^	15.59^aD^				
Neutral detergent fiber (% DM)	Control	63.41^aA^	57.37^B^	56.53^aB^	56.47^aB^	0.695	^**^	^**^	ns
	Cellulase	63.45^abA^	56.52^B^	49.84^bBC^	44.94^bC^				
	*Lactiplantibacillus plantarum*	52.63^b^	49.57	49.91^b^	49.75^ab^				
Acid detergent fiber (% DM)	Control	37.04^abA^	33.13^aB^	33.26^B^	33.27^aB^	0.465	^**^	^**^	^*^
	Cellulase	40.09^aA^	31.01^abB^	28.95^B^	28.00^bB^				
	*Lactiplantibacillus plantarum*	30.86^b^	28.40^b^	27.68	30.36^ab^				
Water-soluble carbohydrates (% DM)	Control	2.55^A^	2.17^B^	0.95^bC^	1.02^C^	0.070	^**^	ns	ns
	Cellulase	2.36^A^	2.22^A^	1.31^aC^	1.64^B^				
	*Lactiplantibacillus plantarum*	3.41^A^	2.36^AB^	1.12^abC^	1.03^C^				

*DM, dry matter; FM, fresh material; SEM, standard error of means*.

* *P < 0.05*;

** *P < 0.01; ns, no significant effect*.

*Values in the same row (^A−C^) or column (^a−c^) followed by different letters differ at P < 0.05*.

### Dynamics of Microbial Populations in *Cassia alata* Silage

The dynamics of LAB, CB, yeasts, and molds are shown in Table 4. The LAB counts significantly increased (*P* < 0.05) followed by a significant decrease (*P* < 0.05) with prolonged ensiling time, and the peak value was observed on day 14. The LAB counts of the three silage groups were 8.06 (CK), 8.27 (CE), and 8.30 (LP) log_10_ CFU/g FM on day 14. The LAB counts of the CE and LP groups increased significantly (*P* < 0.05) compared with the CK group on day 14. The CB counts were highest on day 2 with measurements of 6.54, 5.48, and 5.04. Similarly, the count of yeasts was highest with measurements of 5.60, 5.31, and 5.08 on day 2. The counts of CB and yeasts were undetectable on day 30. Throughout the fermentation process, molds were not detected.

**Table 4:** Microbial population of ensiled *Cassia alata* (log_10_ CFU/g FM).

**Item**	**Treatment**	**Ensiling days**				**SEM**	**Significant**		
		**2**	**6**	**14**	**30**		**Days**	**Treatments**	**Days × Treatments**
Lactic acid bacteria	Control	7.08^cB^	7.45^bB^	8.06^bA^	7.53^B^	0.042	^**^	^**^	ns
	Cellulase	7.38^bB^	8.22^aA^	8.27^aA^	7.68^AB^				
	*Lactiplantibacillus plantarum*	7.45^aC^	8.28^aA^	8.30^aA^	7.76^B^				
Coliform bacteria	Control	6.54^aA^	5.30^aB^	4.80^aC^	ND	0.026	^**^	^**^	^**^
	Cellulase	5.48^bA^	4.15^bB^	3.80^bC^	ND				
	*Lactiplantibacillus plantarum*	5.04^c^	ND	ND	ND				
Yeasts	Control	5.60^aA^	4.74^aB^	3.49^bC^	ND	0.015	^**^	ns	^**^
	Cellulase	5.31^bA^	4.39^bB^	4.02^aC^	ND				
	*Lactiplantibacillus plantarum*	5.08^cA^	4.54^bB^	4.16^aC^	ND				
Molds	Control	ND	ND	ND	ND	–	–	–	–
	Cellulase	ND	ND	ND	ND				
	*Lactiplantibacillus plantarum*	ND	ND	ND	ND				

*FM, fresh material; ND, not detected; SEM, standard error of means; CFU, colony-forming unit*.

^*^P < 0.05;

** *P < 0.01; ns, no significant effect*.

*Values in the same row (^A−C^) or column (^a−c^) followed by different letters differ at P < 0.05*.

### Dynamics of Bacterial Diversity in *Cassia alata* Silage

**Figure 1:**
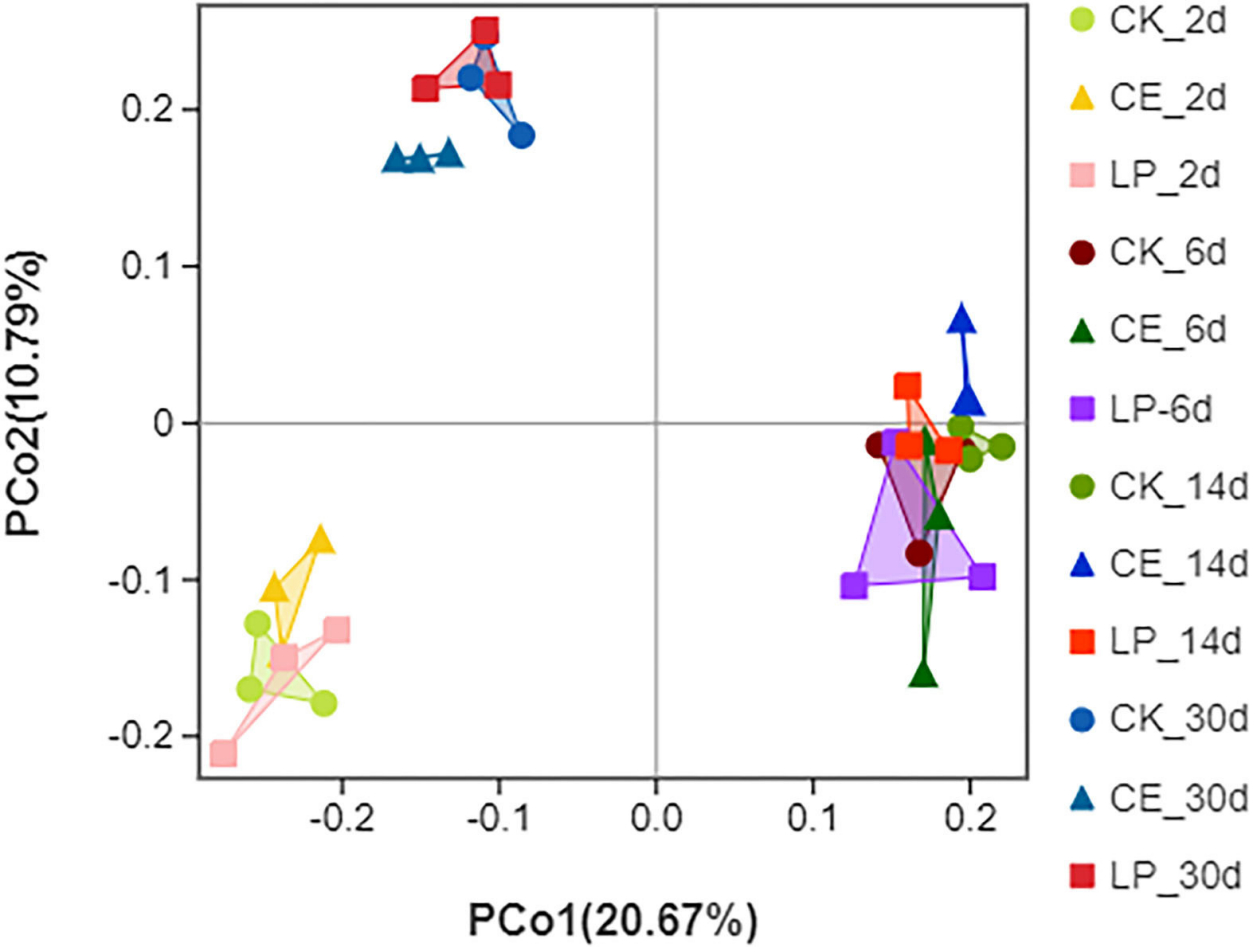
Principal coordinates analysis (PCoA) of bacterial communities for *cassia alata* silage (CK, control; CE, cellulase; LP, *Lactiplantibacillus plantarum*, after 2, 6, 14, and 30 days of ensiling, respectively).

The alpha diversity of dynamic CA silage bacterial communities is shown in Table 5. The good coverage values for all treatments were above 99%, indicating that the sampling data were sufficiently representative of the entire flora of the different samples. The Sob, Chao, and Ace indexes of CE and LP groups decreased significantly (*P* < 0.05) throughout the ensilage period. The result of PCoA is shown in Figure 1. Principal coordinates 1 (PCo1) and 2 (PCo2) explain 20.67 and 10.79% of the total variance in CA silage, respectively. The 2- and 30-day CA silages were clustered in the third and second quadrants, whereas both the 6- and 14-day silages were in the first and fourth quadrants, respectively. All the three groups were significantly separated during different silage times, and then compared with themselves. In addition, the CE group formed a greater degree of separation from the CK group compared with the LP group on day 30.

**Table 5:** Alpha diversity of the bacterial community of ensiled *Cassia alata*.

**Item**	**Treatment**	**Ensiling days**				**SEM**	**Significant**		
		**2**	**6**	**14**	**30**		**Days**	**Treatments**	**Days × Treatments**
Goods-coverage	Control	0.9994^AB^	0.9994^bAB^	0.9993^A^	0.9996^B^	0.00	^**^	ns	ns
	Cellulase	0.9994^B^	0.9993^abA^	0.9994^B^	0.9995^B^				
	*Lactiplantibacillus plantarum*	0.9993^B^	0.9991^aA^	0.9992^B^	0.9995^B^				
Sob	Control	205.67	217.00	190.33	169.67	3.06	^**^	ns	ns
	Cellulase	213.67^B^	230.67^B^	214.33^B^	163.00^A^				
	*Lactiplantibacillus plantarum*	226.67^B^	216.00^B^	213.00^B^	159.00^A^				
Simpson	Control	0.84^A^	0.87^AB^	0.87^bB^	0.87^bAB^	0.00	ns	^**^	ns
	Cellulase	0.86	0.84	0.85^b^	0.85^ab^				
	*Lactiplantibacillus plantarum*	0.85	0.78	0.80^a^	0.83^a^				
Shannon	Control	3.56	3.57^b^	3.66^c^	3.74^b^	0.03	ns	^**^	ns
	Cellulase	3.70^B^	3.47^abAB^	3.38^bA^	3.54^abAB^				
	*Lactiplantibacillus plantarum*	3.42	3.04^a^	3.06^a^	3.36^a^				
Chao	Control	233.10^AB^	243.95^B^	221.38^AB^	192.84^A^	3.33	^**^	ns	ns
	Cellulase	238.21^B^	270.48^C^	243.40^BC^	199.00^A^				
	*Lactiplantibacillus plantarum*	259.13^B^	258.32^B^	260.73^B^	188.96^A^				
Ace	Control	255.68	259.01	233.83^a^	204.13	3.60	^**^	ns	ns
	Cellulase	257.44^B^	280.74^B^	261.05^abB^	197.16^A^				
	*Lactiplantibacillus plantarum*	276.69^B^	280.32^B^	267.45^bB^	197.24^A^				

*CK, control; CE, cellulase; LP, Lactiplantibacillus plantarum; SEM, standard error of means; D, ensiling days effect; T, treatments effect; D **×** T, the interaction effect of treatments and ensiling days*.

^*^P < 0.05;

** *P < 0.01; ns, no significant effect*.

*Values in the same row (^A−C^) or column (^a−c^) followed by different letters differ at P < 0.05*.

### Bacterial Abundance of *Cassia alata* Silage

**Figure 2:**
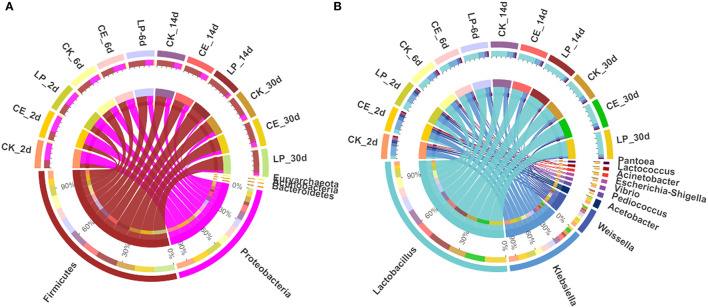
Circos map of bacterial communities at the phylum **(A)** and genus **(B)** levels for *cassia alata* silage (CK, control; CE, cellulase; LP, *Lactiplantibacillus plantarum*, after 2, 6, 14, and 30 days of ensiling, respectively).

**Figure 3:**
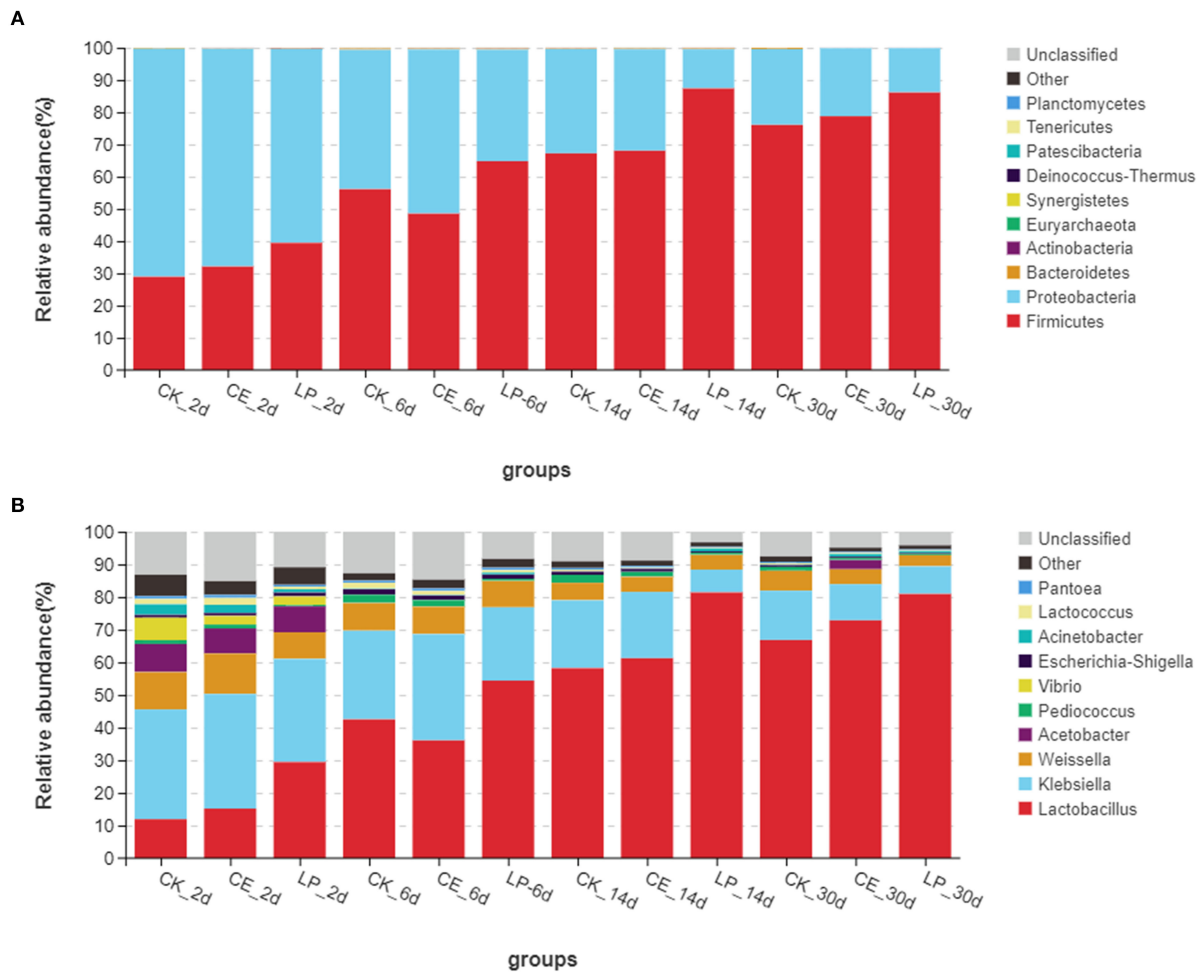
Accumulation map of bacterial communities at the phylum **(A)** and genus **(B)** levels for *cassia alata* silage (CK, control; CE, cellulase; LP, *Lactiplantibacillus plantarum*, after 2, 6, 14, and 30 days of ensiling, respectively).

The relative abundances of bacterial communities of CA silage at the phylum and genus levels are presented in Figure 2 (Circos map) and Figure 3 (accumulation map). Supplementary Tables S1, S2 show the bacterial communities at the top 10 phylum and genus levels of ensiled CA. At the phylum level, *Proteobacteria* was the highest in CK (70.80%), CE (67.58%), and LP (60.19%) groups, and it was the most dominant phylum in CA silage on day 2. However, gradually, *Firmicutes* increased and *Proteobacteria* decreased significantly (*P* < 0.05) as the fermentation proceeded. *Firmicutes* was the most dominant phylum in the CA silage, accounting for 76.07% of CK, 78.76% of CE, and 86.13% of LP on day 30. The relative abundance of *Firmicutes* in the LP group was significantly higher (*P* < 0.05) than in the CK group. After refining the genus level, 31 colonies with relative abundance above 0.1% were detected in the silage. In the early stages of silage, the dominant genus was *Klebsiella*, while the subdominant genera were *Lactobacillus, Weissella*, and *Acetobacter*. Gradually, *Lactobacillus* replaced *Klebsiella* on the 6th, 14th, and 30th day. On day 30, *Lactobacillus* accounted for 66.83% (CK), 72.86% (CE), and 80.94% (LP), respectively. In comparison with the CK group, the relative abundance of *Lactobacillus* in the CE and LP groups were significantly higher (*P* < 0.05), while the relative abundance of *Klebsiella* and *Weissella* in the CE and LP groups significantly decreased (*P* < 0.05).

### Predicted Functions and Pathways of the Bacterial Community in *Cassia alata* Silage

**Figure 4:**
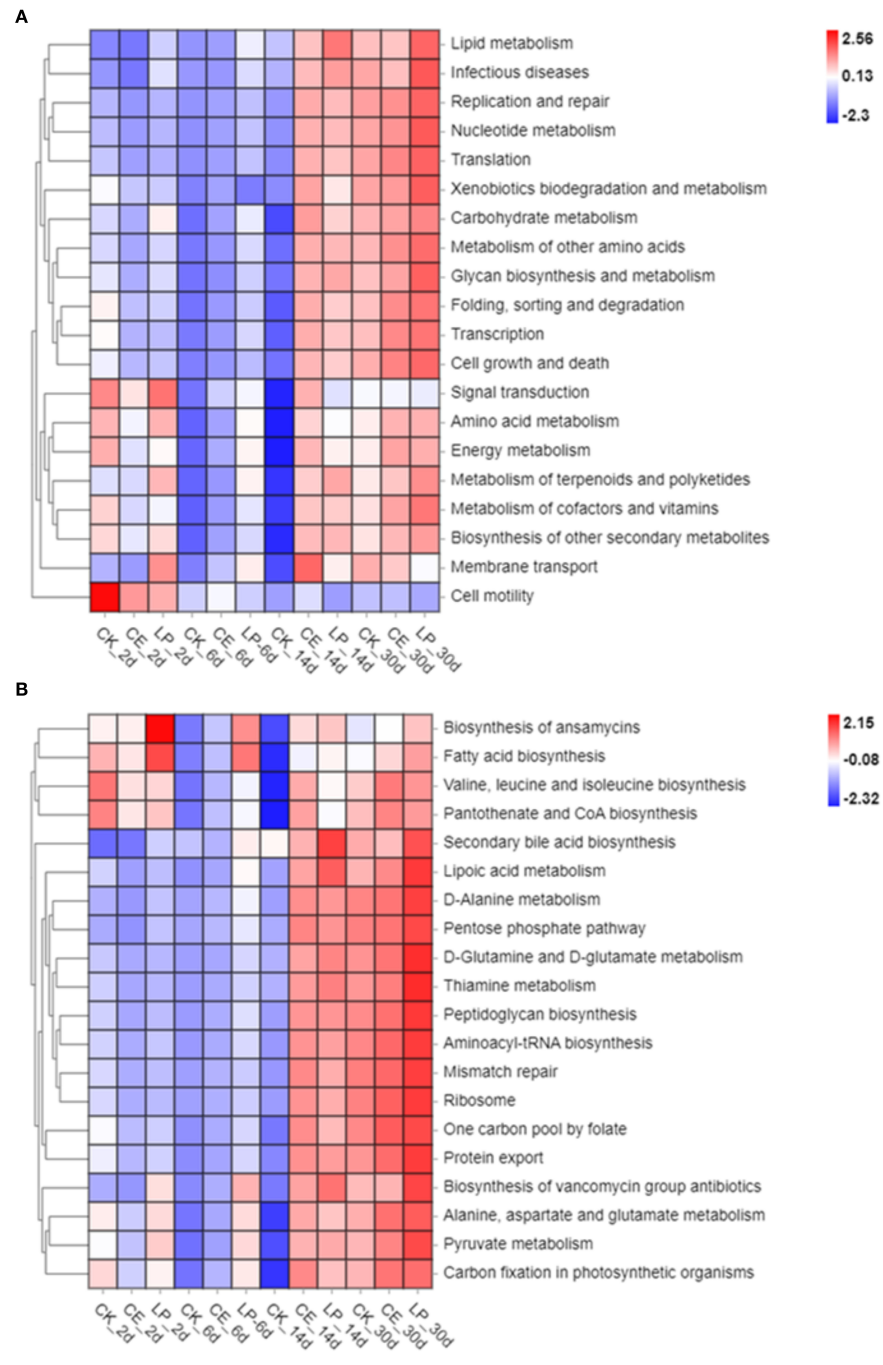
Heatmap of the top 20 predicted functions **(A)** and pathways **(B)** of the bacterial communities analyzed *via* Tax4Fun for *cassia alata* silage (CK, control; CE, cellulase; LP, *Lactiplantibacillus plantarum*, after 2, 6, 14, and 30 days of ensiling, respectively).

The functional and pathway profiles of bacterial colony prediction are shown in Figure 4. The first five predictive functions were carbohydrates metabolism, membrane transport, amino acid metabolism, nucleotide metabolism, and translation. The top five predicted pathways were ABC transporters, a two-component system, purine metabolism, aminoacyl-tRNA biosynthesis, and pyrimidine metabolism. The heatmap of the top 20 predicted functions and pathways implies that the addition of CE and LP might have affected the major functions and pathways.

## Discussion

### Characteristics of Fresh *Cassia alata* Before Ensiling

The moisture content of raw material can affect the growth and reproduction rate of microorganisms during fermentation, and it is an important factor that affects the parameters of silage fermentation (Hu et al., 2009). The DM content in fresh CA was lower than the DM content of 35% required for good silage (Guyader et al., 2018). Therefore, it was necessary to add additives to avoided the problem of massive growth and reproduction of spoilage microbiota, such as *Clostridium*, that might exist in high-moisture silage and facilitated the preparation of high-quality silage for CA (Yitbarek and Tamir, 2014). However, increased NDF and ADF contents might contribute to the ruminant production performance. Silage can effectively reduce NDF and ADF contents and improve the palatability and nutritional value of CA. WSC is an essential substrate for LAB fermentation in the ensilage process. The WSC content of fresh CA was below the ideal content of 6% (Zhang et al., 2019), which was challenging for producing high-quality silage for CA. Therefore, silage additives and CE should be added to promote NDF and ADF hydrolysis to increase the WSC content, and LP should be added to promote LAB colonization, which effectively promotes the fermentation of CA. Collectively, CA was a high-protein forage. However, its disadvantages include high NDF and ADF contents and low WSC content, which hinder the ensilage process without silage additives. Therefore, additions were recommended to improve the quality of CA silage.

### Effects of Cellulase and *Lactiplantibacillus plantarum* on the Fermentation Parameters in *Cassia alata* Silage

The pH value is an important indicator for evaluating the fermentation parameters of silage. During ensilage, the pH values of CE and LP groups were more significant than those in the CK group. Similar to the result of Mu et al. (2020), both CE and LP can decrease the pH value in mixed silage of high-moisture amaranth and rice straw. The pH values of each treatment group initially decreased and then increased during the fermentation. This phenomenon could be caused by the continuous growth and multiplication of *Clostridium butyricum* and the production of butyric acid in low pH and humid conditions, with partial fermentation of lactic acid to butyric acid (He et al., 2020). The lowest pH was observed on day 14, and the pH value of the CE group was below 4.2. Generally, silage also can be considered fermented at a pH below 4.2 (McDonald, 1981). The pH values of the CE group were significantly lower than those of CK and LP groups on day 30. Therefore, CE has a better ability to reduce the pH value of CA silage than LP.

During ensilage, proteins are eventually degraded into amines, amides, and ammonia by the action of plant proteases and microbiota (Kung et al., 2018). Therefore, NH_3_-N can reflect the nutritional value of silage, which is a common indicator of protein hydrolysis (McDonald, 1981). In this trial, the NH_3_-N contents of the CK, CE, and LP groups increased significantly with fermentation. The NH_3_-N contents of the CE and LP groups were significantly lower than those of the CK group. The results of Kung and Shaver (2001) indicated that the action of *Clostridium* and plant protein hydrolases might be the typical cause of NH_3_-N accumulation. Therefore, this phenomenon might have resulted from the effective inhibition of the action of *Clostridium* and plant protein hydrolases by CE and LP. Similar effects on other silage fermentation were achieved by applying CE or LP. The results of Li et al. (2019) show that the addition of CE significantly reduced NH_3_-N in cassava foliage silage. Cheng et al. (2021) found an obvious reduction in NH_3_-N content of paper mulberry treated with B17 (*Lactiplantibacillus plantarum*). Notably, the NH_3_-N content of the LP group was significantly lower than that of the two other groups on the 30th day. Apparently, LP was more conducive to inhibiting proteolysis through the influence of enzymes and microorganisms.

LAB are commonly homofermentative and produce more LA. However, some LAB shift to heterofermentative in forages lacking sugar (Li et al., 2018), thus producing LA and AA (Abdelazez et al., 2022). The LA content of each group increased, and the LP group was the highest on day 30. The AA content of each group remarkably increased during ensilage, of which the CE group had the highest content on day 30. AA can improve the aerobic stability of silage and effectively inhibit fungal reproduction (Schmidt and Kung, 2010). Thus, both LP and CE could promote LAB fermentation. Propionic acid (PA) and butyric acid (BA) reflect the nutritional loss of silage, and both compounds are not conducive to improving the quality of silage, which is undesirable in silage. After day 2, the contents of PA in each group increased significantly with fermentation. The BA content of the CK group remarkably increased during ensilage, indicating that CA ensilage possibly encountered harmful bacteria that multiplied and produced PA and BA. The BA content of the CK group was significantly higher than that of CE and LP group on day 30. Similarly, the results of Guo et al. (2014) showed that CE and LP lowered PA and BA contents compared with the control. Besides, the decrease in BA concentration supported that clostridial fermentation is inhibited by a rapid drop in pH. Therefore, LP and CE can both promote LAB fermentation and inhibit the increase in the number of deterioration bacteria, which can effectively improve the fermentation parameters of silage.

### Effects of Cellulase and *Lactiplantibacillus plantarum* on the Nutrients in *Cassia alata* Silage

High-quality fermentation is accompanied by good preservation of nutrients in the silage. The DM contents among the three groups were lower than the ideal content of 30–35% reported by Guyader et al. (2018). CP content is an indicator for assessing the nutritional value of silage. The contents of CP in CE and LP groups were significantly higher than those of the CK group on day 30. This finding was obtained because both CE and LP facilitated the protection of proteins from catabolism (Li et al., 2017; Mu et al., 2020). NDF and ADF are important indicators of silage quality (Wang et al., 2011). The high contents of NDF and ADF in low-quality silage are not conducive to animal digestion and reduce the digestibility of the feed. With the increase in fiber content, the contents of protein and energy in the silage decreased. Therefore, the lower the fiber content in the feed, the better it is for improving the value of the feed (Xiang-Lin et al., 2008). The contents of NDF and ADF in the CE group were significantly and negatively correlated with ensilage time, and they significantly decreased compared with the CK group on day 30. CE significantly reduces the fiber content, especially NDF and ADF (Guo et al., 2014). WSCs are both decomposition products of plant fibrous materials and fermentation substrates for LAB. The consumption of WSC by LAB decreases the pH, which helps in inhibiting the activity of undesirable microbiota, reducing the loss of nutrients during fermentation, and improving the fermentation parameters of silage. The contents of WSC were significantly higher in the CE group than in the CK and LP groups on day 30. Both the decrease in NDF and ADF contents and the increase in WSC content in the CE group can be attributed to the ability of CE as a cell-wall-degrading enzyme to increase the rate of plant cell-wall degradation and release of glucose (Nadeau et al., 2000). Therefore, CE might be conducive to promoting LAB colonization during ensilage, consistent with the results of Li et al. (2017).

### Effects of Cellulase and *Lactiplantibacillus plantarum* on the Microbial Populations in *Cassia alata* Silage

Microbial communities affect silage quality by affecting the ecosystem in silage (Zhao et al., 2021). LAB plays a leading role in the ensilage process and can produce a large amount of organic acids, which is closely related to the quality of silage (Ni et al., 2015). The LAB counts increase and then decrease during ensilage, reaching the peak value on day 14. This finding was consistent with the change in pH value. It indicates that the effect of the CE and LP groups was better than that of CK group on day 14 (*P* < 0.05), implying that both CE and LP promote LAB growth. Lactobacilli count must reach 10^5^ CFU/g FM for high-quality silage (Cai et al., 1998). The count of LAB reached 8 log_10_ CFU/g FM in the three groups of this trial, indicating that LAB underwent massive multiplication during ensilage, which was beneficial for CA silage quality. CB, which is the main competitor of LAB, hydrolyzed the protein component and decreased the amino acid content, resulting in a loss of nutrients in the silage (Rauramaa et al., 1987). Yeasts, as a type of aerobic microorganisms, deplete oxygen and consume DM as the main flora and convert WSC into ethanol during fermentation (Lv et al., 2020). CB and yeast populations exceeded the limit of 2.00 log_10_ CFU/g FM (Wang et al., 2019b) during fermentation but were undetectable on day 30. Throughout the fermentation process, molds were not detected. CB, yeasts, and molds were not detected, which was beyond our expectations. This finding can be attributed to the continuous multiplication of LAB and the production of LA with a low pH value of the silage.

### Effects of Cellulase and *Lactiplantibacillus plantarum* on the Bacterial Diversity in *Cassia alata* Silage

Microbial community structure, diversity, and function are hot topics in microbial ecology, which have been studied in different media (Wang et al., 2019b; Lv et al., 2020; Zhao et al., 2021). The OTUs (Sob index), diversity (Simpson and Shannon indexes), and richness (Chao and Ace indexes) were studied to estimate the bacterial alpha diversity. The significant changes in alpha diversity among silage periods are caused by the dynamic microbial response to the ensilage process, in which the composition and function of the bacterial flora can obviously vary (Sepehri and Sarrafzadeh, 2019). In the present study, the Sob, Chao, and Ace indexes of CE and LP groups decreased during storage time, indicating that the bacterial diversity in silage decreased. Wang et al. (2020b) confirmed that the addition of CE decreased the Chao and Ace indexes of alfalfa silage flora. Chen et al. (2020b) showed that LP could lower the Ace index of alfalfa silage. Therefore, the addition of CE and LP can reduce the bacterial diversity. This finding was obtained because CE provided more carbon and nitrogen sources for LAB, which promoted the multiplication of LAB. The increase in available nutrients (C, N) promoted LP growth, thereby increasing its proportion in the silage microbial populations, explaining the large LAB numbers observed. LAB produced large amounts of LA and lowered the pH value of silage, thus inhibiting the proliferation of harmful bacteria and decreasing bacterial diversity.

The results of beta diversity reflect the differences in bacterial communities in each individual or treatment (Tian et al., 2021). For further insight into the changes in bacterial communities of CA silage, PCoA of beta diversity was employed. The result of unweighted PCoA could demonstrate the variance in the bacterial community structure of CA silage. The PCoA results show that all three groups were significantly separated during different silage times compared with themselves, indicating obvious changes in bacterial communities. In addition, the CE group formed a greater degree of separation from the CK group compared with the LP group on day 30. Considering that the differences in microbial communities may be responsible for differences in silage quality, CE and LP might help in promoting the growth of different microbial communities, thus enhancing the fermentation parameters, with the CE effect being more remarkable.

### Effects of Cellulase and *Lactiplantibacillus plantarum* on the Bacterial Abundance in *Cassia alata* Silage

The composition of the microbiota is related to silage quality (Tian et al., 2021). Changes in the dominant phylum and genus of CA silage material throughout the ensilage process indicated large changes in the composition and abundance of bacterial communities during ensilage. Therefore, the changes in the fermentation parameters and nutrients of CA are caused by the changes in these specific bacterial communities. On the phylum level, *Proteobacteria* was the most dominant in the early stage of ensilage. *Firmicutes* gradually replaced *Proteobacteria* during ensilage. Mu et al. (2020) reported similar results. After 30 days of ensiling, *Firmicutes* and *Proteobacteria* were the dominant phylum in the mixed silage of high-moisture amaranth and rice straw. Liu et al. (2019) found that *Firmicutes* and *Proteobacteria* were the most dominant phylum, accounting for 99% of the total relative abundance in the late stages of barley silage. Considering that all LAB belong to *Firmicutes* (Tian et al., 2021), with the decrease in pH and increase in LAB during ensilage, *Firmicutes* gradually became the most dominant phylum as the silage time changed. By contrast, *Proteobacteria* gradually decreased. This condition can be explained by the fact that the permeability of the outer membrane of Gram-negative bacteria was affected, thereby inhibiting the growth of *Proteobacteria* (Helander and Mattila-Sandholm, 2000). In conclusion, the addition of both CE and LP contributed to the growth of *Firmicutes* and the inhibition of *Proteobacteria*.

To further study the effect of additives on the bacterial community during ensilage, we examined the bacterial structure of CA silage at the genus level. In the early stages of silage, the dominant genus was *Klebsiella*, and the subdominant genera were *Lactobacillus, Weissella*, and *Acetobacter*. Li et al. (2021) found that the genes involved in NH_3_ formation were mainly classified as encoding nitrite reductase (nirB) and distributed mainly in *Klebsiella*. *Klebsiella* is an acid-intolerant facultative anaerobe that can grow well in anoxic environmental conditions using fermentation substrates (Stewart, 1988). *Klebsiella* is a harmful microorganism in silage; it destabilizes the forage aerobically and causes infectious diseases, such as mastitis (Zadoks et al., 2011). LAB is found in forage crops and silage, many of which isolates had been identified as part of *Lactobacillus* group (You et al., 2021a). Cai et al. (1999) found that *Lactobacillus* is the main microorganism in forage and silage. *Lactobacillus* is the dominant genus in high-quality silage, because it is responsible for driving substrate fermentation during ensilage and plays a crucial role in accumulating LA and lowering pH value (Zi et al., 2021). Some *Lactobacillus* species, such as *Lactiplantibacillus plantarum* and *Lentilactobacillus buchneri*, are commonly used as silage inoculants, because they grow rapidly and inhibit the reproduction of harmful bacteria, which help improve the quality of silage fermentation (Ranjit and Kung, 2000). *Weissella*, an obligately heterofermentative anaerobic bacterium, converts WSC to LA and AA during ensilage fermentation. *Weissella* is another major microorganism in all silages throughout the fermentation process and is an early colonizer (You et al., 2021b). *Acetobacter* is an AA-producing and nitrogen-fixing obligate aerobic bacterium (Pahlow et al., 2015) that usually acts simultaneously with yeast to trigger a metamorphic process in silage (Spoelstra et al., 1988). In barley silages, Liu et al. (2019) found that *Acetobacter* is dominant in the bacterial community once aerobic spoilage occurs. Therefore, as ensilage time changed, *Lactobacillus, Weissella*, and *Acetobacter* continued to produce acid to lower the pH value of the silage, resulting in less *Klebsiella* members. Furthermore, after 30 days of ensilage, *Lactobacillus* remarkably influenced the fermentation of silage by rebuilding the microbiota and became the most dominant genus, thus decreasing the abundance of *Klebsiella, Weissella*, and *Acetobacter*. The significant shift in the bacterial community from *Proteobacteria* to *Firmicutes* during ensilage can be explained by the increase in the total abundance of *Lactobacillus* and *Weissella*, which flourished under the environmental condition that develops during ensilage. Mu et al. (2020) found that the addition of both CE and LP was beneficial to increasing the abundance of *Lactobacillus* in the mixed silage of high-moisture amaranth and rice straw. In this trial, CE and LP further reduced the relative abundance of *Klebsiella* compared with the untreated CK group, suggesting that CE and LP had antibacterial ability against undesirable bacteria. LP group had the highest abundance of *Lactobacillus*. This finding corresponds to the result that both the content of LA and the number of LAB were the highest in the LP group, as mentioned previously. Therefore, LP was more favorable for the rapid accumulation of LA and multiplication of LAB and inhibited the activity of most acid intolerant and aerobic fungi.

### Predicted Functions and Pathways of the Bacterial Community in *Cassia alata* Silage

The functional and pathway prediction of silage microflora was analyzed by Tax4Fun. In comparison with the CK group, the application of both CE and LP increased the abundance of carbohydrate metabolism, membrane transport, amino acid metabolism, nucleotide metabolism, translation, ABC transporters, purine metabolism, aminoacyl-tRNA biosynthesis, and pyrimidine metabolism. The heat maps of predicted functions and pathways imply that the addition of CE and LP might have affected major functions and pathways. Furthermore, the analysis of PCoA and bacterial abundance confirmed that LP plays a pronounced role in the predicted functions and pathways. Therefore, LP greatly influenced the functions of the bacterial community. These results can be attributed to the large changes in some functional bacteria caused by silage additives. However, the exact mechanism remains unclear, thus requiring further studies.

## Conclusion

The study revealed the potential of CA as a raw material for ensilage. Moreover, CE and LP contributed to the fermentation parameters and nutrients of CA silage. The pH value and NH_3_-N content decreased, and the content of CP increased in the silage treated with CE and LP. The NDF and ADF contents were evidently reduced, and WSC content increased upon the addition of CE. The addition of CE and LP reduced bacterial diversity, increased the abundance of *Lactobacillus*, and decreased the abundance of *Klebsiella, Weissella*, and *Acetobacter*. LP had a better effect than CE. In conclusion, CE and LP could further improve the quality of CA silage, and LP had a greater effect on the bacterial community than CE.

## Data Availability Statement

The datasets presented in this study can be found in online repositories. The names of the repository/repositories and accession number(s) can be found in the article/Supplementary Material.

## Author Contributions

YG: conceptualization and project administration. MW and HT: methodology. ZX: software, validation, formal analysis, and data curation. MD: investigation and resources. ZX and JW: writing—original draft preparation. BS and DL: writing—review and editing. YL and GL: supervision. All authors have read and agreed to the published version of the manuscript.

## Funding

This research was supported by the National Nature Science Foundation of China (31872382), the Guangdong Provincial Promotion Project on Preservation and Utilization of Local Breed of Livestock and Poultry (4300-F18260), and the Modern Agricultural Industrial Technology System of Guangdong Province (2019KJ127).

## Conflict of Interest

The authors declare that the research was conducted in the absence of any commercial or financial relationships that could be construed as a potential conflict of interest.

## Publisher's Note

All claims expressed in this article are solely those of the authors and do not necessarily represent those of their affiliated organizations, or those of the publisher, the editors and the reviewers. Any product that may be evaluated in this article, or claim that may be made by its manufacturer, is not guaranteed or endorsed by the publisher.
