# Supplementary material for: Effects of Cellulase and Lactiplantibacillus plantarum on the Fermentation Parameters, Nutrients, and Bacterial Community in Cassia alata Silage
Source: Front Microbiol. 2022 Jul 7;13:926065. doi: 10.3389/fmicb.2022.926065 (PMC9301268; doi:10.3389/fmicb.2022.926065)
Supplement: Supplementary file 1 [file Data_Sheet_1.docx]

**Supplementary Materials:**

**Table S1.** Bacterial communities at the top 10 phylum levels of ensiled *Cassia alata*

| **Item** | **Treatment** | **Ensiling days** | | | | **SEM** | **Significant** | | |
| --- | --- | --- | --- | --- | --- | --- | --- | --- | --- |
|  |  | **2** | **6** | **14** | **30** |  | **Days** | **Treatments** | **Days × Treatments** |
| *Firmicutes* | Control | 28.95^A^ | 56.12^B^ | 67.24^aBC^ | 76.07^aC^ | 1.16 | ** | ** | ns |
|  | Cellulase | 32.12^A^ | 48.54^B^ | 68.06^aC^ | 78.76^aD^ |  |  |  |  |
|  | Lactiplantibacillus plantarum | 39.47^A^ | 64.82^B^ | 87.39^bC^ | 86.13^bC^ |  |  |  |  |
| *Proteobacteria* | Control | 70.80^C^ | 43.26^B^ | 32.37^bAB^ | 23.54^bA^ | 1.15 | ** | ** | ns |
|  | Cellulase | 67.58^D^ | 50.95^C^ | 31.47^bB^ | 21.11^bA^ |  |  |  |  |
|  | Lactiplantibacillus plantarum | 60.19^C^ | 34.60^B^ | 12.16^aA^ | 13.77^aA^ |  |  |  |  |
| *Bacteroidetes* | Control | 0.108 | 0.427 | 0.207 | 0.273 | 0.026 | * | ns | ns |
|  | Cellulase | 0.125^AB^ | 0.291^B^ | 0.256^B^ | 0.040^A^ |  |  |  |  |
|  | Lactiplantibacillus plantarum | 0.120^A^ | 0.330^B^ | 0.280^B^ | 0.027^A^ |  |  |  |  |
| *Actinobacteria* | Control | 0.090 | 0.073 | 0.117 | 0.072 | 0.008 | ns | ns | ns |
|  | Cellulase | 0.113 | 0.116 | 0.078 | 0.059 |  |  |  |  |
|  | Lactiplantibacillus plantarum | 0.154^B^ | 0.148^B^ | 0.123^AB^ | 0.053^A^ |  |  |  |  |
| *Euryarchaeota* | Control | 0.000^A^ | 0.046^B^ | 0.011^AB^ | 0.000^A^ | 0.005 | * | ns | ns |
|  | Cellulase | 0.000 | 0.033 | 0.075 | 0.000 |  |  |  |  |
|  | Lactiplantibacillus plantarum | 0.000^A^ | 0.023^B^ | 0.015^AB^ | 0.000^A^ |  |  |  |  |
| *Synergistetes* | Control | 0.008 | 0.009 | 0.007 | 0.000 | 0.001 | * | ns | ns |
|  | Cellulase | 0.005^AB^ | 0.009^B^ | 0.008^B^ | 0.000^A^ |  |  |  |  |
|  | Lactiplantibacillus plantarum | 0.003^A^ | 0.005^AB^ | 0.008^B^ | 0.000^A^ |  |  |  |  |
| *Deinococcus-Thermus* | Control | 0.000 | 0.008 | 0.002 | 0.010 | 0.001 | ns | ns | ns |
|  | Cellulase | 0.001 | 0.005 | 0.002 | 0.007 |  |  |  |  |
|  | Lactiplantibacillus plantarum | 0.005 | 0.001 | 0.001 | 0.002 |  |  |  |  |
| *Patescibacteria* | Control | 0.000 | 0.001 | 0.015 | 0.000 | 0.001 | ns | ns | ns |
|  | Cellulase | 0.001^AB^ | 0.004^AB^ | 0.010^B^ | 0.000^A^ |  |  |  |  |
|  | Lactiplantibacillus plantarum | 0.000 | 0.001 | 0.000 | 0.000 |  |  |  |  |
| *Tenericutes* | Control | 0.000 | 0.006 | 0.005 | 0.000 | 0.001 | ** | ns | ns |
|  | Cellulase | 0.000^A^ | 0.005^B^ | 0.002^AB^ | 0.000^A^ |  |  |  |  |
|  | Lactiplantibacillus plantarum | 0.000^A^ | 0.009^B^ | 0.001^AB^ | 0.000^A^ |  |  |  |  |
| *Planctomycetes* | Control | 0.000 | 0.001 | 0.000 | 0.004 | 0.001 | ns | ns | ns |
|  | Cellulase | 0.003 | 0.000 | 0.000 | 0.003 |  |  |  |  |
|  | Lactiplantibacillus plantarum | 0.008^A^ | 0.003^AB^ | 0.000^B^ | 0.000^B^ |  |  |  |  |
| *Other* | Control | 0.004 | 0.004 | 0.003 | 0.004 | 0.001 | ** | ns | ns |
|  | Cellulase | 0.002^A^ | 0.016^B^ | 0.008^AB^ | 0.007^AB^ |  |  |  |  |
|  | Lactiplantibacillus plantarum | 0.007^A^ | 0.020^B^ | 0.007^A^ | 0.003^A^ |  |  |  |  |
| *Unclassified* | Control | 0.048^B^ | 0.044^B^ | 0.018^A^ | 0.020^A^ | 0.001 | ** | ns | ns |
|  | Cellulase | 0.049^C^ | 0.039^C^ | 0.027^B^ | 0.010^A^ |  |  |  |  |
|  | Lactiplantibacillus plantarum | 0.047^C^ | 0.038^BC^ | 0.022^AB^ | 0.017^A^ |  |  |  |  |

SEM, standard error of means. **P < 0.05*; ***P < 0.01*; ns, no significant effect; values in the same row (^A−C^) or column (^a−c^) followed by different letters differ at *P < 0.05*.

**Table S2.** Bacterial communities at the top 10 genus levels of ensiled *Cassia alata*

| **Item** | **Treatment** | **Ensiling days** | | | | **SEM** | **Significant** | | |
| --- | --- | --- | --- | --- | --- | --- | --- | --- | --- |
|  |  | **2** | **6** | **14** | **30** |  | **Days** | **Treatments** | **Days × Treatments** |
| *Lactobacillus* | Control | 11.93^aA^ | 42.51^abB^ | 58.22^aC^ | 66.83^aC^ | 0.92 | ** | ** | ns |
|  | Cellulase | 15.20^aA^ | 36.08^aB^ | 61.25^aC^ | 72.86^bD^ |  |  |  |  |
|  | Lactiplantibacillus plantarum | 29.45^bA^ | 54.40^bB^ | 81.41^bC^ | 80.94^cC^ |  |  |  |  |
| *Klebsiella* | Control | 33.63^C^ | 27.17^BC^ | 20.77^bAB^ | 15.12^bA^ | 0.60 | ** | ** | ns |
|  | Cellulase | 35.11^A^ | 32.56^A^ | 20.32^bB^ | 11.07^aC^ |  |  |  |  |
|  | Lactiplantibacillus plantarum | 31.58^C^ | 22.45^B^ | 6.98^aA^ | 8.45^aA^ |  |  |  |  |
| *Weissella* | Control | 11.42^B^ | 8.46^AB^ | 5.36^A^ | 6.11^bA^ | 0.26 | ** | * | ns |
|  | Cellulase | 12.39^C^ | 8.41^B^ | 4.61^A^ | 4.55^abA^ |  |  |  |  |
|  | Lactiplantibacillus plantarum | 8.17^B^ | 8.06^B^ | 4.50^A^ | 3.43^aA^ |  |  |  |  |
| *Acetobacter* | Control | 8.64^B^ | 0.02^A^ | 0.01^A^ | 0.01^A^ | 0.34 | ** | ns | ns |
|  | Cellulase | 7.69^B^ | 0.02^A^ | 0.05^A^ | 2.83^A^ |  |  |  |  |
|  | Lactiplantibacillus plantarum | 7.96^B^ | 0.01^A^ | 0.03^A^ | 0.01^A^ |  |  |  |  |
| *Pediococcus* | Control | 1.17^bA^ | 2.53^bB^ | 2.46^cB^ | 1.16^bA^ | 0.06 | ** | ** | * |
|  | Cellulase | 1.18^bAB^ | 1.98^bC^ | 1.46^bBC^ | 0.65^aA^ |  |  |  |  |
|  | Lactiplantibacillus plantarum | 0.33^aA^ | 0.68^aB^ | 0.68^aB^ | 0.41^aAB^ |  |  |  |  |
| *Vibrio* | Control | 6.90^B^ | 0.008^A^ | 0.001^A^ | 0.004^A^ | 0.29 | ** | ns | ns |
|  | Cellulase | 2.64^B^ | 0.027^A^ | 0.002^A^ | 0.008^A^ |  |  |  |  |
|  | Lactiplantibacillus plantarum | 2.69 | 0.004 | 0.005 | 0.004 |  |  |  |  |
| *Escherichia-Shigella* | Control | 0.84^A^ | 1.67^B^ | 0.98^A^ | 0.55^bA^ | 0.07 | ** | ns | ns |
|  | Cellulase | 0.89^B^ | 1.39^C^ | 0.96^B^ | 0.38^abA^ |  |  |  |  |
|  | Lactiplantibacillus plantarum | 1.19 | 1.26 | 0.39 | 0.29^a^ |  |  |  |  |
| *Acinetobacter* | Control | 3.23^bB^ | 0.19^A^ | 0.08^A^ | 0.17^A^ | 0.108 | ** | ns | ** |
|  | Cellulase | 2.52^abB^ | 0.17^A^ | 0.12^A^ | 0.80^A^ |  |  |  |  |
|  | Lactiplantibacillus plantarum | 1.02^a^ | 0.61 | 0.85 | 0.54 |  |  |  |  |
| *Lactococcus* | Control | 1.63^bB^ | 1.79^B^ | 0.51^bA^ | 0.36^A^ | 0.05 | ** | ** | ** |
|  | Cellulase | 2.10^cC^ | 1.18^B^ | 0.24^aA^ | 0.29^A^ |  |  |  |  |
|  | Lactiplantibacillus plantarum | 0.73^aB^ | 0.73^B^ | 0.20^aA^ | 0.19^A^ |  |  |  |  |
| *Pantoea* | Control | 0.87^B^ | 0.63^AB^ | 0.50^bA^ | 0.42^bA^ | 0.02 | ** | * | ns |
|  | Cellulase | 0.91^B^ | 0.79^B^ | 0.51^bA^ | 0.37^abA^ |  |  |  |  |
|  | Lactiplantibacillus plantarum | 0.58^AB^ | 0.76^B^ | 0.28^aA^ | 0.26^aA^ |  |  |  |  |

SEM, standard error of means. **P < 0.05*; ***P < 0.01*; ns, no significant effect; values in the same row (^A−C^) or column (^a−c^) followed by different letters differ at *P < 0.05*
